# Assessment and report of individual symptoms in studies of delirium in postoperative populations: a systematic review

**DOI:** 10.1093/ageing/afae077

**Published:** 2024-04-18

**Authors:** Emily M L Bowman, Aoife M Sweeney, Danny F McAuley, Chris Cardwell, Joseph Kane, Nadine Badawi, Nusrat Jahan, Halla Kiyan Iqbal, Callum Mitchell, Jessica A Ballantyne, Emma L Cunningham

**Affiliations:** Centre for Public Health, Queen’s University Belfast, Block B, Institute of Clinical Sciences, Royal Victoria Hospital site, Grosvenor Road, Belfast BT12 6BA, Northern Ireland; Centre for Experimental Medicine, Queen’s University Belfast, Wellcome-Wolfson Institute for Experimental Medicine, 97 Lisburn Road, Belfast BT9 7BL, Northern Ireland; Centre for Public Health, Queen’s University Belfast, Block B, Institute of Clinical Sciences, Royal Victoria Hospital site, Grosvenor Road, Belfast BT12 6BA, Northern Ireland; Centre for Experimental Medicine, Queen’s University Belfast, Wellcome-Wolfson Institute for Experimental Medicine, 97 Lisburn Road, Belfast BT9 7BL, Northern Ireland; Centre for Public Health, Queen’s University Belfast, Block B, Institute of Clinical Sciences, Royal Victoria Hospital site, Grosvenor Road, Belfast BT12 6BA, Northern Ireland; Centre for Public Health, Queen’s University Belfast, Block B, Institute of Clinical Sciences, Royal Victoria Hospital site, Grosvenor Road, Belfast BT12 6BA, Northern Ireland; Centre for Public Health, Queen’s University Belfast, Block B, Institute of Clinical Sciences, Royal Victoria Hospital site, Grosvenor Road, Belfast BT12 6BA, Northern Ireland; Centre for Public Health, Queen’s University Belfast, Block B, Institute of Clinical Sciences, Royal Victoria Hospital site, Grosvenor Road, Belfast BT12 6BA, Northern Ireland; Centre for Public Health, Queen’s University Belfast, Block B, Institute of Clinical Sciences, Royal Victoria Hospital site, Grosvenor Road, Belfast BT12 6BA, Northern Ireland; Centre for Public Health, Queen’s University Belfast, Block B, Institute of Clinical Sciences, Royal Victoria Hospital site, Grosvenor Road, Belfast BT12 6BA, Northern Ireland; Centre for Public Health, Queen’s University Belfast, Block B, Institute of Clinical Sciences, Royal Victoria Hospital site, Grosvenor Road, Belfast BT12 6BA, Northern Ireland; Centre for Public Health, Queen’s University Belfast, Block B, Institute of Clinical Sciences, Royal Victoria Hospital site, Grosvenor Road, Belfast BT12 6BA, Northern Ireland

**Keywords:** delirium, symptoms, postoperative delirium, cognition, attention, systematic review, older people

## Abstract

**Objectives:**

Delirium is most often reported as present or absent. Patients with symptoms falling short of the diagnostic criteria for delirium fall into ‘no delirium’ or ‘control’ groups. This binary classification neglects individual symptoms and may be hindering identification of the pathophysiology underlying delirium. This systematic review investigates which individual symptoms of delirium are reported by studies of postoperative delirium in adults.

**Methods:**

Medline, EMBASE and Web of Science databases were searched on 03 June 2021 and 06 April 2023. Two reviewers independently examined titles and abstracts. Each paper was screened in duplicate and conflicting decisions settled by consensus discussion. Data were extracted, qualitatively synthesised and narratively reported. All included studies were quality assessed.

**Results:**

These searches yielded 4,367 results. After title and abstract screening, 694 full-text studies were reviewed, and 62 deemed eligible for inclusion. This review details 11,377 patients including 2,049 patients with delirium. In total, 78 differently described delirium symptoms were reported. The most reported symptoms were inattention (*N* = 29), disorientation (*N* = 27), psychomotor agitation/retardation (*N* = 22), hallucination (*N* = 22) and memory impairment (*N* = 18). Notably, psychomotor agitation and hallucinations are not listed in the current Diagnostic and Statistical Manual for Mental Disorders-5-Text Revision delirium definition.

**Conclusions:**

The 78 symptoms reported in this systematic review cover domains of attention, awareness, disorientation and other cognitive changes. There is a lack of standardisation of terms, and many recorded symptoms are synonyms of each other. This systematic review provides a library of individual delirium symptoms, which may be used to inform future reporting.

## Key Points

- Current binary classifications of delirium neglect description of its individual symptoms.
- This is likely hindering understanding and management of delirium syndrome and its underlying biology.
- In studies of postoperative delirium, 78 differently-described symptoms were found.
- There are high levels of heterogeneity in the methods of assessing and reporting delirium.
- Many of the symptoms reported in postoperative delirium studies are not included in the Diagnostic and Statistical Manual of Mental Disorders, Fifth Edition, Text Revision (DSM-5-TR).

## Background

Delirium is, most commonly, considered as present or absent,as assessed using validated screening tools [1, 2], based on accepted diagnostic criteria [3]. Reasonably, these tools and criteria focus on the core symptoms—such as inattention and altered consciousness/arousal—considered necessary to constitute a delirium syndrome. There are, however, a broader range of symptoms that can occur as part of a delirium and which can have significant impacts and implications for patients’ experiences, long-term health and clinical care [4–7]. Given the current absence of identifiable and treatable mechanisms underlying delirium symptoms [8], accurate identification and measuring of symptoms are necessary if treatable mechanisms underlying these symptoms are to be discovered [9–14], and calls have been made to integrate the underlying mechanisms of delirium, i.e. the acute encephalopathy, with its clinical features [10, 12]. The reward for such efforts, it is hoped, will be the identification of specific delirium symptoms as an indicator of particular underlying mechanisms. This would lead on to the identification of treatable traits, for example, by performance of data-driven cluster analyses of specific symptoms with biomarkers or imaging data [15], and the discovery and validation of novel therapeutics for delirium. This approach has been successful in studies of other clinical syndromes such as Acute Respiratory Distress Syndrome, which have utilised analysed large datasets combining clinical and biomarker data to identify links between underlying mechanisms and clinical features [16].

When delirium is reported as simply present or absent, it is likely that patients with symptoms falling short of the diagnostic criteria for delirium are included in ‘no delirium’ or ‘control’ groups in studies, potentially compromising analyses. The phenotype of subsyndromal delirium describes a condition falling on a continuum between no delirium and delirium defined by the Diagnostic and Statistical Manual for Mental Disorders [17, 18], but it does not specify which symptoms are present [19–21].

Attempts have been made in research studies to categorise delirium symptoms by psychomotor subtype i.e. hyperactive, hypoactive or mixed; however, this neglects description of individual delirium symptoms and is not sufficient to facilitate in-depth investigation of how specific mechanisms give rise to specific symptoms. Recent publications have called for comprehensive reporting of delirium symptoms [4, 5, 9, 10, 22–24], and phone applications have been developed by Tieges et al. and Hall et al. to improve monitoring of attention [25] and levels of arousal, respectively [26]. Variations in performance in tests of inattention have been reported [27], as have differential outcomes dependant on altered arousal [28]. The Delirium Subtyping Initiative recently highlighted the lack of standardisation of clinical features across studies [11].

This review included only studies from postoperative settings as surgical populations have one of the highest incidence rates of delirium [29, 30]. The ability to plan studies and delirium assessments for a specific short postoperative period also makes this population a useful model to study delirium.

This systematic review aims to address the key component of improving specificity in delirium research, by assessing which individual symptoms of delirium are being reported in postoperative populations, and how often they are reported. Therefore, the study question is: Which individual symptoms of delirium are reported by studies of postoperative delirium in adults?

## Methods

The protocol of this review was written and conducted in accordance with the Preferred Reporting Items for Systematic Reviews and Meta-Analyses Protocols checklist [31] and the PRISMA 2020 guidelines [32]. This systematic review was registered prospectively with PROSPERO, registration CRD42021236622 [33].

### Study types

This systematic review included both observational and intervention studies of hospital patients undergoing surgery of any type, which assessed for postoperative delirium, and reported individual delirium symptoms. Articles that did not investigate postoperative delirium, individual delirium symptoms, or use a validated delirium screening tool were excluded. Case reports, case series, editorials, reviews and systematic reviews were excluded. References of the excluded systematic reviews were screened for relevant papers.

### Participant types

Included participants were adults over the age of 18. Studies investigating only or a majority of patients with pre-existing cognitive impairments such as dementia, pre-operative delirium, Wernicke’s encephalopathy and neurological disorders such as Parkinson’s disease were excluded. Also excluded were studies investigating alcohol abuse or withdrawal, brain tumours and aneurysms. Patients with pre-existing cognitive impairments were excluded to increase the likelihood that the reported symptoms resulted from the syndrome under scrutiny—delirium—and not due to other conditions over which delirium may have been superimposed.

### Setting and exposure

All included participants in this review were exposed to surgery of any type and were assessed for postoperative delirium, using a validated tool, and individual symptoms of delirium.

### Outcome measures

The primary outcome is the reporting and frequency of any individual symptoms of delirium during the seven days following any type of surgery. The reported symptoms will be presented in alignment with the current Diagnostic and Statistical Manual of Mental Disorders, Fifth Edition, Text Revision (DSM-5-TR) criteria [34–37].

Within included studies, the delirium must have been measured by a delirium screening tool that is validated against DSM criteria for delirium [34–37], and the delirium must have been reported alongside individual symptoms. Examples of such screening tools include, but are not limited to, the Confusion Assessment Method (CAM) [38], the Short-CAM [39] or the 4 ‘As’ Test [40].

The secondary outcomes included the reporting of delirium severity or psychomotor subtypes alongside individual delirium symptoms.

### Search strategy and selection criteria

Full details of the search strategy are detailed in the Supplementary Material. Search terms for delirium and its characteristics, as well as ‘postoperative’ were included. Medline, EMBASE and Web of Science databases were first searched on 03 June 2021. The search strategy can be found in Supplementary Table T1a–c.

After removal of duplicated results, two reviewers (E.B., A.S.) independently examined the resultant titles and abstracts. The full text of the potentially relevant studies was retrieved. Due to the nature of the study question and lack of information required to answer the study question in the abstracts, we expected many full-text studies to require screening. The review team was thus expanded to include eight reviewers: three senior reviewers (E.B., E.L.C. and A.S.), and five junior reviewers (N.B., N.J., H.K.I., C.M. and J.B.). Each individual attended an online meeting explaining an outline of the topic, the research question and systematic review procedures for reviewers. This also provided the opportunity for reviewers to ask questions. A list of reviewers and the screening guide can be found in Supplementary Table T2a and b. Each paper was screened in duplicate, with at least one primary reviewer screening each paper. Each reviewer recorded their decisions and reasons on an individual excel data sheet, provided in advance. Conflicts in decisions were settled by consensus discussion involving all three senior reviewers. Reasons for study exclusion were included in the PRISMA systematic review flow chart.

### Data extraction

Data extraction was completed by the lead reviewer (E.B.). Each study was listed by title, first author, year and study type. Data extracted from each study included the following information: surgical population, total population N, delirium population N, delirium %, method of delirium diagnosis, time/frequency of delirium screening, personnel carrying out delirium screening, other assessment tools used, time/frequency of other assessments, primary study aims, individual symptoms reported, severity reported, severity measurement tool, psychomotor subtypes reported yes/no.

The data extracted from each study was synthesised quantitatively and narratively to capture the types and frequency of symptoms reported in included studies.

All included studies were quality assessed for risk of bias. Details of the methods and results of these assessments can be found in the Supplementary Material.

## Results

### Identification of studies

**Figure 1:**
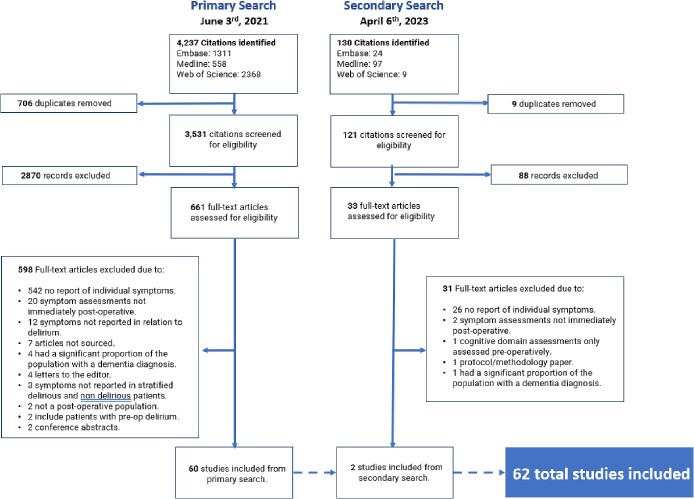
PRISMA flow chart.

The results of the search are detailed in Figure 1. In total, over the two searches, 62 full-text studies were included in this review [4, 26, 41–98].

### Study characteristics

This systematic review of 62 studies consisted of 12,024 participants including 2,235 participants with delirium. The characteristics of included studies are shown in Table 1**.** When accounting for studies of same cohorts, this review included 11,377 participants, 2,049 of which had delirium. Of note, the reported number of participants with delirium is the maximum number of delirium occurrences from each study, as there were occurrences where delirium incidences changed depending on the postoperative day, without disclosing how many new deliriums or delirium resolutions had occurred. Furthermore, the reported total number of delirium occurrences accounts for only full diagnoses of delirium or ‘acute confusional state’ and does not include subsyndromal delirium diagnoses. Two studies reported subsyndromal delirium, one of which did not specify exact incidence [46], and one which reported 12 cases [85]. Many of the included studies did not disclose who conducted the delirium assessments. In studies which provided such information, the personnel were usually physicians, nurses, trained researchers, psychiatrists or a combination. The results of the study quality assessments can be located in Supplementary Figures S2 and S3, and Supplementary Table T3.

**Table 1:** A table detailing the characteristics of included studies. *N* = number.

**Characteristic**	**Studies No. (*N* = 62)**
Publication date, range, years	1996–2023
***Countries in which study was conducted, N***	18
Japan	10 [53, 59, 60, 64, 70, 71, 73, 74, 78, 90]
United States of America	10 [46, 47, 54, 56, 85, 86, 89, 93, 94, 99]
United Kingdom	8 [4, 26, 41, 44, 67–69, 82]
Netherlands	4 [45, 61, 79, 91]
Turkey	4 [76, 80, 95, 110]
Canada	2 [42, 96]
Denmark	2 [63, 83]
France	2 [84, 98]
Greece	2 [77, 81]
South Korea	2 [57, 65]
Germany	2 [92, 111]
China	1 [75]
New Zealand	1 [55]
Norway	1[58]
Portugal	1 [51]
Russia	1 [72]
Taiwan	1 [66]
***Study design***	
Prospective observational cohort	37 studies [41, 42, 44, 45, 48–50, 52, 54–57, 59, 61, 62, 66–69, 72, 74, 76–79, 82–86, 89, 92, 94, 96, 98, 99, 111]
Retrospective cohort	9 [53, 60, 63–65, 73, 87, 88, 90]
Intervention	5 [70, 81, 93, 95, 97]
Validation	4 [43, 51, 71, 75]
Correlation	2 [46, 47]
Cross-sectional	2 [80, 91]
Case–control	1 [26]
Qualitative	1 [58]
Longitudinal	1 [4]
***Surgery type***	
Elective Orthopaedic Emergency Orthopaedic	20 [43, 44, 46–50, 56, 57, 60, 62, 67–69, 75, 80–82, 85, 93] 12 [4, 26, 45, 48–50, 65, 70, 76, 80, 85, 97]
Cardiac Non-Cardiac	18 [41, 42, 52, 58, 61, 72, 78, 79, 83, 87–89, 91, 92, 95, 96, 99, 111] 5 [54, 66, 77, 86, 94]
Gastro-intestinal	5 [59, 63, 75, 82, 90]
Urological	5 [55, 75, 81, 82, 99]
Vascular	4 [55, 81, 82, 99]
Abdominal	3 [71, 98, 99]
General	3 [43, 55, 85]
Gynaecological	3 [55, 81, 99]
Plastic/dermatologic	2 [55, 64]
Surgical ICU	2 [51, 75]
Thoracic	2 [75, 99]
Ambulatory	1 [84]
Breast	1 [90]
Head and Neck	1 [53]
Haematology/oncology	1 [85]
Medical	1 [85]
Neurosurgery	1 [43]
Non-emergency	1 [54]
Pancreatic	1 [74]
Hepatobiliary	1[99]
Spinal	1 [73]
Total participants, range, *N*	10–1,608
Total participants with delirium, range, *N*	1–200

The frequency of published studies meeting inclusion criteria showed an increasing trend over time until 2022, which can be seen in Supplementary Figure S1.

### Primary outcome: Reported delirium symptoms

This systematic review found 78 differently described symptoms of delirium. The mean number of symptoms reported by each study was 5.69, range 1–16. Table 2 shows how the reported symptoms may be categorised according to the current DSM-5-TR criteria, and how many times the symptom was reported in the included papers. Table 3 displays the reported symptoms which cannot be categorised using the current DSM-5-TR criteria. The large volume of ‘other additional disturbances in cognition’ was categorised into general, behavioural, mood-related, motor, sleep, physical and other neuropsychiatric symptoms. Adequate categorisation of these symptoms was challenging due to the lack of definition of many symptoms. The five most reported symptoms were inattention (*N* = 29), disorientation (*N* = 27), psychomotor agitation/retardation (*N* = 22), hallucination (*N* = 22) and memory impairment (*N* = 18).

**Table 2:** A table detailing the symptoms reported in this systematic review, categorised under the current DSM-5-TR guidelines. . . *N* = number.

**Section**	**Symptoms in the Diagnostic and Statistical Manual for Mental Disorders–5–Text revision (DSM-5-TR) description of delirium**	**Similar symptoms reported in literature**	***N* Studies**	**References**
1	Disturbance in attention and concentration	Concentration	6	[43, 50, 59, 70, 82, 92]
		Inattention	29	[19, 42, 44–47, 49, 51, 54, 55, 61, 63, 65–67, 73–76, 78, 80, 82, 83, 87, 91, 92, 97, 111, 112]
		Registration	1	[74]
		Calculation	1	[74]
		Visual attention	2	[48, 94]
2	Reduced awareness and arousal	Alertness	2	[76, 80]
		Altered level of consciousness	14	[44–47, 49, 51, 54, 55, 61, 75, 83, 84, 87, 97]
		Arousal	5	[19, 21, 51–53]
		Awareness	3	[47, 56, 93]
		Coma	1	[74]
		Confusion	5	[46, 57, 59, 74, 93]
		‘*Excitement’*	1	[88]
		Lucidity	1	[60]
		Sleepiness/somnolence	3	[46, 74, 82]
		Stupor	1	[74]
3	Acute change	Acute onset	8	[44, 45, 54, 55, 61, 75, 83, 87]
		Latency time	1	[50]
		Temporal onset	3	[76, 78, 80]
4	Fluctuation	Fluctuation	12	[43–45, 49, 54, 61, 63, 75, 80, 82, 83, 87]
		Variability of symptoms	2	[76, 78]
5A	Memory deficit	Memory impairment	18	[42, 43, 46, 47, 50, 54–56, 59, 63, 76, 78, 80, 91, 92, 94, 111]
		Recall	2	[74, 94]
5B	Disorientation	Disorientation	27	[63, 64] [74–76, 78–80, 85, 88, 90, 99] [41, 43, 46, 47, 49, 82, 93, 95] [50–52, 54, 55, 58]
5C	Language	Difficulty communicating	1	[48]
		Inappropriate communication/language/speech	16	[41–43, 47, 49, 58, 63, 64, 68, 74–76, 80, 82, 85, 92]
		‘*Incoherence*’	1	[43]
		*‘Command of information’*	1	[76]
5D	Visuospatial ability	Visuospatial ability	4	[42, 43, 63, 80]
5E	Perception	*‘Change of reality’*	1	[48]
		Hallucination	22	[41, 43, 49–51, 56, 58, 62–64, 95] [69, 75–77, 79, 83]
		Illusion	5	[41, 50, 64, 75, 83]
		Pareidolia	1	[62]
		Perceptual disturbance	12	[46, 47, 51, 54, 55, 63, 71, 76, 78, 80, 82, 92]

**Table 3:** A table detailing the symptoms reported in this systematic review, which could not be categorised under the current DSM-5-TR criteria. Symptoms were allocated as behavioural, mood-related, physical or other neuropsychiatric symptoms other than perceptual disturbances. *N* = number.

**Symptoms categories not listed in DSM-5-TR**	**Symptoms not listed in DSM-5-TR**	***N* Studies**	**References**
Cognitive impairment	Cognitive impairment	6	[56, 74, 76, 90, 112, 113]
Executive function	Executive function	1	[42]
Behavioural symptoms and manifestations	Agitation	9	[42, 43, 50, 53, 79, 81, 83, 95, 96]
	Aggressiveness	4	[47, 83, 93, 96]
	Failure to co-operate	1	[93]
	‘*Inappropriate behaviour’*	9	[46, 57, 58, 64, 71, 75, 82, 85, 112]
	‘*Inappropriate conduct*’	1	[41]
	Shouting/yelling	2	[57, 93]
	‘Trying to remove catheter or intravenous line.’	2	[62, 63]
	Urgent calls for attention	2	[47, 48]
	Verbal/physical abuse	1	[93]
	Restlessness	2	[83, 93]
	Self-destruction	1	[93]
	Roaming	1	[62]
	Contradictions	1	[48]
	*‘Dramatic scenes’*	1	[48]
	Distress	1	[80]
Mood-related symptoms	Affective symptoms	1	[43]
	Euphoria	1	[83]
	Anxiety	3	[47, 83, 111]
	Depression	1	[62]
	‘*Emotional*’/lability of mood	11	[46, 48, 50, 56, 59, 63, 76, 78, 80, 83, 86]
	Feelings after delirium	1	[48]
	*‘Inappropriate mood’*	1	[49]
	Irritable	1	[83]
Physical and functional symptoms	Dependency in ADL	1	[59]
	Dependency in mobility	1	[59]
	Falling from bed	1	[62]
	Hypokinesia	1	[43]
	Physical disorder	4	[56, 63, 76, 80]
	Praxis	1	[74]
	Somatic illness	1	[43]
Psychomotor symptoms	Psychomotor Agitation/Retardation	22	[41, 46, 47, 49–51, 53–55, 62–64, 75, 76, 78, 83–86, 90, 91, 93]
Sleep symptoms	Sleep/wake cycle disturbance	14	[43, 46, 49, 54, 55, 57–59, 62, 63, 76, 78, 80, 84]
	Insomnia	1	[72]
	Nightmares	2	[57, 62]
Other neuropsychiatric (other than perceptual disturbances)	Delusion	7	[43, 49, 63, 68, 76, 79, 80]
	Disorganised thinking	11	[44–46, 53–55, 61, 75, 83, 87, 97]
	Psychosis	2	[49, 56]
	Psychotic symptoms	3	[50, 68, 85]
	Suspiciousness	1	[47]
	Paranoia	2	[68, 95]
	Thought content disorder	1	[78]
	‘*Disturbed Thought process*’	2	[63, 68]
	‘*Misconception*’	2	[50, 68]

### Primary outcome: Reporting of delirium symptoms In those with and without delirium

In studies which reported delirium symptoms with respect to delirium and no delirium groups, the following symptoms occurred more often in delirium participants than non-delirium participants: inattention [41, 44, 78, 96], perceptual disturbance [78, 96], disorientation [43, 78], improper conduct [43], inappropriate communication [43], illusions [43], hallucinations [43, 78, 79], delusions [78], nightly confusion [48], sleep disturbance [61, 78], cognitive change [78], lability of mood [78], variability of symptoms [78] and motor/verbal behaviour [78]. Also seen were impairments in motor functioning [43, 78, 96], central processing speed [41, 44, 96], verbal fluency [96], logical and verbal memory [41, 44, 96] and executive function [41, 44]. A report of a prospective study stratified symptoms by treatment group, but not by delirium and non-delirium groups [73].

### Primary outcome: Ascertainment and description of symptoms

Methods and scales used for delirium diagnosis were described in varying levels of detail across included studies. In total, 25 delirium diagnosis tools were used in the included studies, with delirium diagnosis being supported, or cognition further assessed, by 55 cognitive assessment methods. Supplementary Table TS4 lists the delirium assessment tools used in the included studies. The 25 delirium diagnostic tools included several variations of tools such as the CAM, the Delirium Rating Scale (DRS) and the Diagnostic and Statistical Manual of Mental Disorders (DSM). The most frequently used delirium screening tool was the CAM [4, 26, 41, 44–47, 52, 56–58, 60, 63, 67–69, 73, 77, 79, 82, 85, 86, 89, 94, 98, 99].

Table 4 shows the measures of relevant symptoms that were used in conjunction with or in addition to the delirium assessments. The mini mental state examination was the most used cognitive assessment [26, 41, 44–46, 48–50, 62, 65, 67–70, 72, 75, 78, 80, 81, 85, 87–89, 92, 96, 98].

**Table 4:** The cognitive assessment tools used in included studies, and how many studies used each tool.

**Tests for relevant symptoms**	**Frequency**
***Cognitive/dementia screening tools***	
Mini Mental State Examination (MMSE)	28
Clock drawing test	3
Cognitive Drug Research computerised assessment system (simple reaction time, digit vigilance, choice reaction time)	3
Digit symbol test	2
Mini-cog	2
Montreal cognitive assessment (MoCA)	2
Abbreviated MMSE	1
Standardised Mini-Mental Test (SMMT)	1
Executive Clock Drawing Test (CLOX) 1	1
CLOX 2	1
Hasegawa’s dementia scale (HDS-R).	1
Informant Questionnaire on Cognitive Decline in the Elderly (IQCODE)	1
***Agitation and sedation***	
Richmond Agitation Sedation Scale (RASS)	9
The Short Portable Mental Status Questionnaire (SPMSQ)	1
Modified RASS (mRASS)	1
***Global scales and measures***	
Clinical dementia rating scale	3
Gottfries-Bråne-Steen scale	1
***Attention and concentration***	
Days of the week and months of the year backwards	5
The grooved pegboard test	1
Time to print the alphabet	1
***Consciousness and arousal***	
Observational Scale of Level of Arousal (OSLA)	2
Glasgow coma scale	2
***Memory***	
Digit span test	3
Trail making test A and B	3
Colour trails	2
Wechsler logical memory test	2
Word list learning task	2
Delayed recall test	1
New York paragraph recall test	1
Benton visual retention	1
Mattis-kovner verbal recall	1
Mattis-kovner verbal recognition	1
Rey auditory verbal learning test	1
Rey-osterrieth complex figure	1
***Premorbid intelligence***	
National Adults Reading Test (NART)	2
***Verbal fluency***	
Letter and category fluency tasks	2
Semantic verbal fluency test	2
Ammons quick test for verbal IQ	1
Animal naming	1
Controlled word association	1
Fluid cognition measured using tests of verbal fluency	1
***Executive/Frontal Function***	
Stroop Test	2
The symbol digit modalities test	2
Battery of frontal dysfunction	1
***Personality traits***	
Big Five Inventory (BFI)	1
***Functioning***	
Modified blessed dementia scale	1
***Visuospatial***	
Noise Pareidolia Test (NPT)	1
***Psychotic experiences***	
Questionnaire of Psychotic Experiences (QPE)	1

### Secondary outcomes: Reporting of severity and psychomotor subtypes

Delirium severity was reported in nine studies [4, 26, 43, 45, 52, 65, 66, 73, 99]. Four of these studies assessed severity using the DRS (DRS-R-98) [4, 26, 45, 65], two used the Nursing Delirium Screening Scale (NuDESC) [43, 66], one assessed severity by determining its ‘magnitude’ based on the Organic Brain Syndrome Scale [52], one used the CAM-Severity and CAM-ICU-7 [99] and one study did not report their methods of determining severity [73]. Psychomotor subtypes of delirium were reported in 12 studies [43, 52, 53, 55, 57, 68, 70, 85–88, 91], yet only one described the individual symptoms observed within the subtypes [52].

## Discussion

This systematic review assessed which individual symptoms of delirium are reported in studies of postoperative delirium. Across 62 included studies [4, 26, 41–98], with 11,377 participants, including 2,049 participants with delirium, 78 differently-described symptoms of delirium were reported. The five most reported symptoms were inattention, disorientation, psychomotor agitation, hallucination and memory impairment.

To our knowledge, this review is the first to investigate the number and consistency of reporting methods of individual delirium symptoms in the postoperative setting. This review highlights the extent of variation in clinical presentation of delirium, and its many means of description, that may previously have been underappreciated. Many of the symptoms reported in this review are synonyms of one another, synonyms for those defined in the DSM-5-TR or go unmentioned in the DSM-5-TR. The lack of consistency and standardisation in description of the symptoms may limit the comparison and combination of data from delirium studies.

Furthermore, it is noteworthy that the largest number of symptoms reported in Tables 2 and 3 would fall into the ‘other additional disturbance in cognition’ category of the DSM-5-TR, indicating that this diagnostic manual does not fully encompass the syndrome phenotype. Many of these symptoms might be considered the most disturbing of delirium, such as change in command of information, paranoia, sleep cycle disturbances, depression, physical disorder, agitation and aggressiveness. In 2023, the ICD-11 describes delirium similarly to the DSM-5-TR, with the addition of examples of behavioural symptoms like agitation, restlessness and impulsivity [100]. While the ICD-11 is slightly more inclusive, neither manual covers all symptoms catalogued by this systematic review. Until now, delirium has been operationalised for assessment and improving clinical detection; however, this is often at the expense of specialist characterisation. Future modifications to these diagnostic manuals to ensure complete and comprehensive descriptions of individual components of the syndrome may lead to improved standardisation of reporting across studies.

Many of the reported symptoms occurred in patients not diagnosed with delirium, emphasising the importance of looking beyond a binary delirium diagnostic threshold. Koster et al. show that ‘no delirium’ participants also had symptoms of memory problems, concentration problems, confusion, sleep disturbance, dependency in activities of daily living (ADL) and mobility and emotional problems [61]. Although not significant, a greater proportion of the no delirium group actually had confusion and dependency in ADL [61]. Also of note, Ottens et al. found 77.3% of the participants with hallucinations did not develop postoperative delirium [79]. In addition, Bryson et al. found that testing cognition with the Clock drawing test did not distinguish between delirium and non-delirium [42]. However, many included studies did not describe the symptoms which existed in the non-delirium group. Reasons for this likely vary—a number of included studies were recording individual symptoms of delirium to validate one assessment tool against another, hence only focussing on those with delirium. It is likely that many studies have the data for those without a delirium diagnosis, and have not reported it, or perhaps did not have room in their publications to do so. Furthermore, these findings suggest that, if delirium endotypes are identified, the endotypes can also be explored at individual-symptom levels.

In conjunction with the heterogeneity of reported symptoms, this review also found that 25 different delirium diagnosis tools and 48 different cognitive tests were used. The variation in delirium assessment tools can only increase levels of heterogeneity between studies. However, the vast number of cognitive assessment tools used displays potential in ways for the field to properly assess fully defined symptoms, using the broad toolbox of cognitive tests that are available.

It is also notable that out of 3,652 studies screened, only 62 met inclusion criteria, showing a large gap in individual symptoms reported in most studies, despite the fact that the validated tools used assess such domains. Recent literature encourages the concept of delirium as a disorder, encompassing a clinical syndrome, its precipitants and the underlying pathophysiological disturbances [101]. Within this disorder, it is likely that there are subtypes of delirium, depending on the clinical features, aetiologies and mechanisms that are taking place. Identification of these subtypes will require standardisation of definitions as well as large, big-data driven statistical analyses combining clinical features and biomarker data [11, 22]. The lack of standardisation shown by this review makes current comparison and combination of studies difficult.

The commonly used delirium assessment tools include many key delirium symptoms, so it is probable that most delirium studies possess symptom data for delirium, subsyndromal and non-delirium groups. There was a noteworthy disconnect between the methods and results of the papers that were excluded during the full-text review stage. The methods sections of many articles describe detailed delirium symptom assessment methods but continue to report delirium as a dichotomous outcome within their results.

### Future implications

The current heterogeneity of delirium symptom reporting found by this systematic review demonstrates the need for more consistent recording and reporting of individual symptoms beyond those captured by standard delirium screening tools. Building on the successes in delirium research thus far, achieving more accurate phenotyping will facilitate the identification of ‘endotypes’, where the phenotype is linked with its underlying biological mechanism. Future studies can then conduct complex data analyses using data of individual symptoms, clustered with biomarker data, yielding insights beyond those possible using binary yes/no delirium classifications. By meticulously and accurately recording delirium symptoms, a record of potential traits to be targeted by future treatments is created. Moreover, if such traits can be associated with delirium biomarkers, pharmacological treatments can be trialled and personalised to individuals.

### Strengths and limitations

This systematic review has several strengths. It followed PRISMA 2020 guidelines and involved a large literature search with strict search criteria, inclusion and exclusion criteria. The large volume of full-text papers requiring review called for use of an expanded review team, within which all reviewers received full training, all papers were screened in duplicate, all papers were screened by at least one senior reviewer and conflicts were resolved in consultation with a third reviewer. Although the review included only postoperative populations, a vast range of surgery types were represented, from 18 different countries, including many participants. This review also included studies of high quality according to the NOS, RoB2 and ROBINS-I assessments. In addition, although the primary search was carried out in June 2021, a secondary search was completed just before review completion in April 2023, to ensure inclusion of all relevant and most recent studies.

We acknowledge several potential limitations of this systematic review. There is a lack of consistency of terminology across the literature, therefore it is possible that despite our use of broad MeSH terms, not all studies reporting delirium symptoms in postoperative populations were captured. The heterogeneity of included studies and nature of the review question limited us to synthesising the results narratively, rather than by meta-analysis. Although summarising the results qualitatively may provide some insight into the proportions of commonly occurring symptoms and heterogeneity, we cannot provide information relating to the statistical significance of our results. Furthermore, we did not use PsychInfo for database searches due to the postoperative target population, but this may have introduced a bias in which symptoms were detected. The review question was restricted to postoperative populations; however, repetition of this review in other populations where delirium is incident, such as critical care and general medicine, is required. Several papers that reported on individual symptoms of delirium or cognitive domains were excluded from this study because they did not assess immediately after surgery. Instead, assessments were completed pre-operatively [102], after 7 days [103–107], 6 months [108] or 1 year [109]. Of note, we did not consider use of antipsychotics as a symptom of interest [98]**.** It is likely that included studies recorded data for other symptoms other than those reported, and excluded studies may have recorded individual symptoms that they did not report.

Due to the large number of symptoms reported, often with low frequency, the heterogeneity in terms used to describe symptoms, and the low number of studies reporting biomarkers and severity, only simple quantitative analyses were deemed appropriate. Future studies, with sufficient data, could consider subgroup or cluster analyses to investigate associations of individual delirium symptoms with factors such as surgery type, biomarkers, severity and type of anaesthesia.

## Conclusion

This systematic review highlights the lack of standardisation in symptom definition and recording methods in the literature with 62 studies of postoperative patients, reporting 78 symptoms of delirium. A large proportion of reported symptoms fall without current DSM-5-TR categories, highlighting a lack of specificity in these guidelines, often for some of the most traumatic symptoms of delirium.

Furthermore, this study highlights the variation in delirium assessment methods, cognitive assessment tests and psychomotor subtype reporting across the included studies. We provide further evidence that delirium is most often reported as present or absent as 90.3% of full-text studies screened were excluded due to not reporting individual symptoms. Delirium remains without recommended pharmacological treatment, and its underlying pathophysiological mechanisms remain largely as hypotheses. Combination of individual symptom reports with physiological data, such as biomarker levels measured from biospecimens, may allow for identification of delirium subtypes. This systematic review provides a library of reported individual delirium symptoms from postoperative cohorts, which may be used to inform future studies of delirium symptoms, its underlying biology and potential methods for identifying targeted treatments.

## Supplementary Material

aa-23-2074-File002_afae077
