# Supplementary material for: Assessment and report of individual symptoms in studies of delirium in postoperative populations: a systematic review
Source: Age Ageing. 2024 Apr 18;53(4):afae077. doi: 10.1093/ageing/afae077 (PMC11028403; doi:10.1093/ageing/afae077)
Supplement: aa-23-2074-File002_afae077 [file aa-23-2074-file002_afae077.docx]

**Assessment and report of individual symptoms in studies of delirium in postoperative populations: a systematic review**

***Supplementary Materials***

Table of Contents

[Section 1 – Search Strategy 2](#_Toc162003214)

[Supplementary Table T1a – Search strategy for Embase 2](#_Toc162003215)

[Supplementary Table T1a – Search strategy for Medline 3](#_Toc162003216)

[Supplementary Table T1a – Search strategy for Web of Science 3](#_Toc162003217)

[Section 2 – Reviewer Team and Screening Instructions 5](#_Toc162003218)

[Supplementary Table T2a - Table detailing the full text review team and their roles. 5](#_Toc162003219)

[Supplementary Table T2b - Screening instructions provided to all reviewers. 5](#_Toc162003220)

[Section 3 – Study Years 8](#_Toc162003221)

[Supplementary Figure S1 - A graph displaying the number of included studies reported in each year between 1996 and 2023 8](#_Toc162003222)

[Section 4 – Risk of Bias and Study Quality 9](#_Toc162003223)

[Supplementary Methods and Results for Risk of Bias Assessments 9](#_Toc162003224)

[Supplementary Figure S2 - A traffic light plot and weighted bar plot showing the results from the risk of bias assessment using the revised tool to assess risk of bias in randomized trials (RoB2). 9](#_Toc162003225)

[Supplementary Material S3 - A traffic light plot and weighted bar plot showing the results from the risk of bias assessment using the tool to assess risk of bias in non-randomized trials (ROBINS-I). 10](#_Toc162003226)

[Supplementary Table T3 - The results of risk of bias assessments in cohort and case control studies using the Newcastle Ottawa Scale (NOS). 10](#_Toc162003227)

[Section 5 – Delirium Assessment Tools 12](#_Toc162003228)

[Supplementary Table T4 - The delirium assessment tools used in included studies, and how many studies used each tool. 12](#_Toc162003229)

#

# Section 1 – Search Strategy

Search terms for disorders associated with delirium and its characteristics were used including altered mental status, acute mental status change, encephalopathy, acute confusion, acute brain syndrome, acute brain failure, acute psycho-organic syndrome, acute organic psychosyndrome, psychotic disorders, mental processes, and coma. Search terms for ‘postoperative’ included post-operative and after surgery. Terms used to search for symptoms included ‘delirium symptoms’, attention, inattention, awareness, cognitive change, cognitive dysfunction, cognitive impairment, disorganised thinking, disorganized thinking, consciousness, altered state of consciousness, arousal, disorientation, hallucination, neurobehavioural manifestations and behavioural symptoms.

## Supplementary Table T1a – Search strategy for Embase

|  | **Embase Search Terms** | **Results** |
| --- | --- | --- |
| 1 | exp delirium/ or deliri$.ti,ab or Altered mental status.mp.or acute mental status change.mp. or Encephalopathy.mp. or (acute adj2 (confusion$ or "brain syndrome" or "brain failure" or "psycho-organic syndrome" or "organic psychosyndrome")).mp. or psychotic disorders.mp or mental processes.mp or coma/ | 174812 |
| 2 | Post?operat*.mp. or after surgery | 1260079 |
| 3 | Exp symptom/ or attention/ or inattention.mp.or awareness/ or cognitive change.mp. or cognitive dysfunction.mp. or cognitive impairment.mp. or disorganized thinking.mp. or disorganised thinking.mp. or consciousness/ or "altered state of consciousness"/ or arousal/ or disorientation/ or hallucination/ or neurobehavioural manifestations.mp or behavioral symptoms.mp | 597679 |
| 4 | 1 and 2 and 3 | 1894 |
| 5 | Limit 4 to humans and embase | 1311 |

## Supplementary Table T1a – Search strategy for Medline

|  | **Medline Search Terms** | **Results** |
| --- | --- | --- |
| 1 | exp delirium/ or deliri$.ti,ab or Altered mental status.mp.or acute mental status change.mp. or Encephalopathy.mp. or Coma/ or Psychotic Disorders/ or Mental Processes/ or (acute adj2 (confusion$ or "brain syndrome" or "brain failure" or "psycho-organic syndrome" or "organic psychosyndrome")).mp. | 148118 |
| 2 | Post-operative or postoperative or after surgery | 962125 |
| 3 | Symptom.mp. or attention/ or inattention.mp.or awareness/ or cognitive change or cognitive dysfunction or cognitive impairment.mp. or disorganized thinking.mp. or disorganised thinking.mp. or consciousness/ or altered state of consciousness.mp or arousal/ or disorientation.mp. or hallucinations.mp. or exp Hallucinations/ or neurobehavioural manifestations.mp. or Behavioral Symptoms/ | 449185 |
| 4 | 1 and 2 and 3 | 643 |
| 5 | Limit 4 to humans | 558 |

## Supplementary Table T1a – Search strategy for Web of Science

|  | **Web of Science using topics**  **Search Terms** | **Results** |
| --- | --- | --- |
| **1** | TOPIC: (Deliri*) OR TOPIC: ("Acute confusion") OR TOPIC: ("Altered mental status") OR TOPIC: ("Acute mental status change") OR TOPIC: ("Acute brain failure") OR TOPIC: (encephalopathy) OR TOPIC: (coma) OR TOPIC: ("psychotic disorder") OR TOPIC: ("mental processes") OR TOPIC: ("acute brain syndrome") OR TOPIC: ("acute psycho-organic syndrome") OR TOPIC: ("acute organic psychosyndrome") | 124,534 |
| **2** | TOPIC: (post$operative*) OR TOPIC: (after surgery) | 832,669 |
| **3** | TOPIC: (symptom) OR TOPIC: (attention) OR TOPIC: (inattention) OR TOPIC: (awareness) OR TOPIC: ("cognitive change") OR TOPIC: ("cognitive dysfunction") OR TOPIC: ("cognitive impairment") OR TOPIC: ("disorganised thinking") OR TOPIC: ("disorganized thinking") OR TOPIC: (consciousness) OR TOPIC: ("altered state of consciousness") OR TOPIC: (arousal) OR TOPIC: (disorientation) OR TOPIC: (hallucination) OR TOPIC: ("neurobehavioural manifestations") OR TOPIC: ("behavioral symptoms") | 2,409,458 |
| **4** | TOPIC: (animal*) OR TOPIC: (child*) | 3,118,362 |
| **5** | #1 AND #2 AND #3 NOT #4 | 2,368 |

# Section 2 – Reviewer Team and Screening Instructions

## Supplementary Table T2a - Table detailing the full text review team and their roles.

| **Reviewer Name** | **Review Role** | **Position** |
| --- | --- | --- |
| Emily Bowman | Lead reviewer, primary reviewer | PhD researcher |
| Emma Cunningham | Primary reviewer | Clinical lecturer, consultant geriatrician |
| Aoife Sweeney | Primary reviewer | Postdoctoral researcher |
| Callum Mitchell | Secondary reviewer | Masters of Public Health student |
| Halla Kiyan Iqbal | Secondary reviewer | Masters of Public Health student |
| Jessica Ballantyne | Secondary reviewer | Masters of Public Health student |
| Nadine Badawi | Secondary reviewer | Masters of Public Health student |
| Nusrat Jahan | Secondary reviewer | Masters of Public Health student |

## Supplementary Table T2b - Screening instructions provided to all reviewers.

**Assessments and reports of individual symptoms of delirium in postoperative populations: a systematic review**

*Thank you for agreeing to contribute to the full text screening of this systematic review. This is a large scale, novel endeavour which requires input from a large review team. All credits will be given in publication. For full information on the review please visit the Prospero application:* <https://www.crd.york.ac.uk/prospero/display_record.php?ID=CRD42021236622>

*If you have any questions, please do not hesitate to contact me at* [***ebowman01@qub.ac.uk***](mailto:ebowman01@qub.ac.uk)***.***

*Please read these instructions in full before commencing review.*

***Subject Context***

Delirium is a clinical syndrome that is characterised by an alteration in the awareness and cognition of patients, with acute and fluctuating onset. This is a result of pathophysiological disruption and an insult such as infection, surgery, brain damage or many other events. Current classification methods of delirium are not effective in describing the full patient profile, as they either classify delirium as one type, or describe it based on psychomotor disturbance (hypoactive, hyperactive or mixed). We want to find out which individual symptoms are reported in postoperative delirium studies, rather than simply reporting on the presence of the syndrome.

***Aims of Systematic Review***

1. Identify studies of postoperative delirium patients (Any surgery type) that report individual delirium symptoms.
2. Identify which symptoms are reported and if there are patterns in these symptoms.
3. Note if these studies also report on delirium severity, and if this is associated with certain symptoms.

***Instructions for full text screening***

- In the Microsoft Team files you will find a folder allocated to your name. Within each folder is your allocated papers for screening.
- Please read each paper fully to identify those that adhere to the inclusion criteria (Inclusion and exclusion criteria can be found below).
- Download your individual ‘Screening Proforma’ and note your decision for each paper that you screen**- Include, Exclude, Maybe** and note a **Reason**.
- In included papers, highlight the sections which include your reason for including the paper.
- We may ask you for clarification on your reasons for excluding or including papers.
- When your decisions are received and collated, you may need to discuss conflicting decisions or ‘maybe’ decisions with those who also read the same paper.
- Where conflicting decisions are not resolved in duplicate, a third reviewer will contribute.
- When screening a non-English language paper, I recommend using the Google Translate app to scan the paper using your phone camera. This will instantly translate it for you.
- You may need to source supplementary materials for the paper to clarify if the study is fit for inclusion- this can usually be found online.

***Inclusion Criteria***

- Studies of patients in hospital undergoing surgery of any type and who develop delirium after any surgery. **They must report individual delirium symptoms.**

**Examples of individual symptoms:**

**Altered level of consciousness** (might be reported using Alert, Voice, Pain, Unresponsive scale (AVPU), Glasgow Coma Score (GCU), The Observational Scale of Level of Arousal (OSLA) or other measure/comment)

**Inattention** (Might be reported subjectively or using an objective test such as Months of the Year Backwards (MOTYB) or Mini Mental State Examination (MMSE).

**Disorganised Thinking** (might be tested subjectively or by asking basic questions or using the Abbreviated Mental Test-4 (AMT4)

**Alertness** (Reported subjectively or using an objective measurement)

Note- This list is not exhaustive.

The most common delirium diagnostic tools are the Confusion Assessment Method (CAM) and the 4AT, but there are many others too. Some studies will simply report the test used and whether delirium is present or not- these should not be included in the review. However, if the study provides a breakdown of the test for individual patients, and how they scored in each domain, this is of interest to us.

- Study types: All clinical trials, randomised controlled trials, observational studies, and qualitative studies including interventional design studies;
- All languages;
- All timeframes;
- Adults aged 18 and over (no paediatric studies)

***Exclusion Criteria***

- Articles which do not report individual symptoms;
- Articles not investigating postoperative delirium;
- Animal studies;
- Studies investigating only children (under 18s);
- Patients with pre-existing dementia;
- Patients who were delirious pre-operatively.
- Wernicke’s encephalopathy;
- Alcohol abuse/withdrawal;
- Brain tumours;
- Aneurysms;
- Parkinson’s disease

# Section 3 – Study Years

Supplementary Figure S1 - A graph displaying the number of included studies reported in each year between 1996 and 2023.

# Section 4 – Risk of Bias and Study Quality

## Supplementary Methods and Results for Risk of Bias Assessments

The Revised Tool to Assess Risk of Bias in Randomised Trials (RoB2) [41], and Risk Of Bias In Non-Randomized Studies- of Interventions (ROBINS-I) tools were used [42]. The data from the RoB2 and ROBINS-I assessments was visualised using the Robvis tool [43]. The Newcastle-Ottawa Quality Assessment Scale (NOS) was used to appraise each non-randomised study [44], all performed by EB.

Risk of bias was assessed using the RoB2 in three studies [74,85,97], for which the results are shown in **Supplementary Figures S2**. Two studies had overall low concerns of risk of bias [74,97], and one study had some concerns [85]. The ROBINS-I was used to assess two studies, which showed low risk of bias [99], **Supplementary Figure S3**. Fifty-eight studies were assessed using the NOS [4,26,45–73,75–84,86,88–96,98,100,102,103,105,106], of which the average score was 8.21 out of 9 and the range was 6-9. The scores of these studies are shown in **Supplementary Table T3.**

##
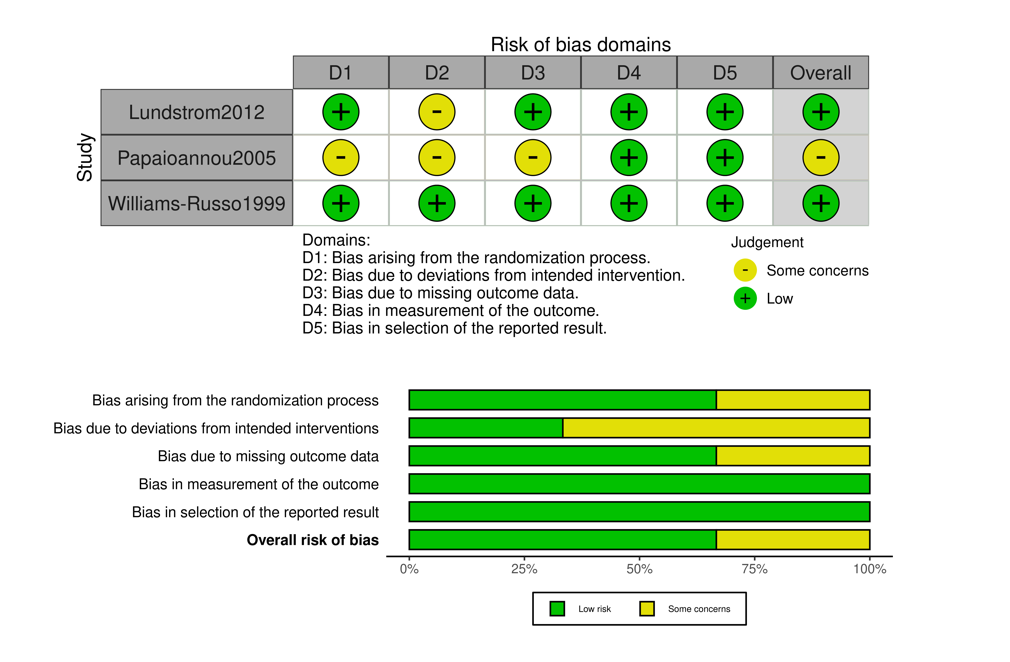
Supplementary Figure S2 - A traffic light plot and weighted bar plot showing the results from the risk of bias assessment using the revised tool to assess risk of bias in randomized trials (RoB2).

##
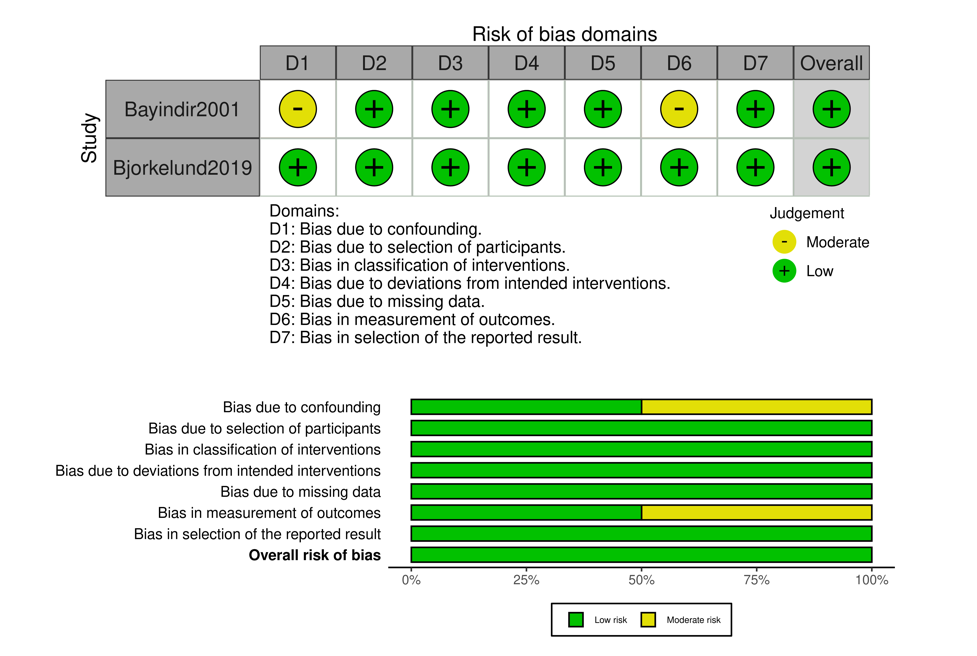
Supplementary Material S3 - A traffic light plot and weighted bar plot showing the results from the risk of bias assessment using the tool to assess risk of bias in non-randomized trials (ROBINS-I).

## Supplementary Table T3 - The results of risk of bias assessments in cohort and case control studies using the Newcastle Ottawa Scale (NOS).

| First Author | Score |
| --- | --- |
| Abelha2011 | 9 |
| Andersson2001 | 9 |
| Arizumi2020 | 8 |
| Aya2019 | 8 |
| Benoit2005 | 9 |
| Brouquet2019 | 9 |
| Brown2011 | 8 |
| Bryson2011 | 8 |
| Cinar2019 | 9 |
| Cunningham2017 | 8 |
| De Jonghe2007 | 9 |
| Denny2020 | 9 |
| Denny2017 | 9 |
| Duppils2000 | 9 |
| Duppils2004 | 9 |
| Duppils2007 | 7 |
| Eriksson2002 | 8 |
| Guay2023 | 9 |
| Guenther2021 | 8 |
| Hall2020 | 9 |
| Hasegawa2015 | 8 |
| Hesse2019 | 9 |
| Hight2018 | 9 |
| Hirsch2016 | 9 |
| Hong2018 | 8 |
| Instenes2018 | 7 |
| Kaneko1997 | 8 |
| Kijima2020 | 8 |
| Koster2009 | 6 |
| Kurbegovic2015 | 8 |
| Kuwahara2008 | 8 |
| Kyeong2018 | 8 |
| Lin2016 | 9 |
| Lowery2007 | 8 |
| Lowery2008 | 7 |
| Lowery2010 | 8 |
| Maeda2019 | 8 |
| Medvedeva2021 | 8 |
| Mizuno2018 | 8 |
| Mu2020 | 8 |
| Mutlu2018 | 8 |
| Ntalouka2020 | 8 |
| Ohki2006 | 8 |
| Ottens2020 | 8 |
| Ozbas2018 | 9 |
| Partridge2019 | 6 |
| Pedersen2014 | 8 |
| Rice2011 | 9 |
| Shim2015 | 8 |
| Smulter2015 | 8 |
| Smulter2019 | 6 |
| Tan2008 | 9 |
| Tieges2013 | 9 |
| Tsuitsui1996 | 7 |
| Van Dellen2014 | 9 |
| Walzer1997 | 8 |
| Youngblom2014 | 9 |
| Average | 8.21 |

# Section 5 – Delirium Assessment Tools

## Supplementary Table T4 - The delirium assessment tools used in included studies, and how many studies used each tool.

| **Delirium Diagnosis Tool** | **Frequency** |
| --- | --- |
| Confusion Assessment Method (CAM) | 27 |
| CAM for the Intensive Care Unit (CAM-ICU) | 7 |
| CAM-ICU-7 severity scale | 1 |
| CAM for non-intubated patients (CAM-IMC) | 1 |
| CAM-Severity (CAM-S) | 1 |
| 3-Minute Diagnostic Interview for CAM-defined Delirium Chinese version (3D-CAM-CN) | 1 |
| Family CAM (FAM-CAM) | 1 |
| Delirium rating scale, revised, 1998 (DRS-R-98) | 7 |
| Japanese Delirium Rating Scale (DRS-J) | 1 |
| Korean Delirium Rating Scale (KDRS) | 1 |
| Delirium symptom interview (DSI) | 1 |
| Diagnostic and Statistical Manual of Mental Disorders**-4** (DSM-4) | 11 |
| DSM-III | 4 |
| DSM-III-Text Revision (DSM-III-TR) | 1 |
| DSM-IV-Text Revision (DSM-IV-TR) | 1 |
| DSM-5 | 1 |
| Nursing Delirium Screening Scale (NuDESC) | 5 |
| Intensive care delirium screening checklist (ICDSC) | 3 |
| Modified Organic Brain Syndrome Scale (MOBS) | 3 |
| Organic brain syndrome scale (OBS) | 3 |
| Delirium observation screening (DOS) scale | 2 |
| The Delirium Index (DI) | 2 |
| The 4 'A's Test (4AT) | 2 |
| Edinburgh Delirium test box (EDTB) | 1 |
| International Classification of Diseases 10th revision (ICD-10) | 1 |
| Memorial Delirium Assessment Scale (MDAS) | 1 |
| The Neelon and Champagne (NEECHAM) Confusion Scale | 1 |
| Delirium assessment method not described | 3 |
